# pH-Dependent Extraction of Antioxidant Peptides from Red Seaweed *Palmaria palmata*: A Sequential Approach

**DOI:** 10.3390/md22090413

**Published:** 2024-09-10

**Authors:** Sakhi Ghelichi, Ann-Dorit Moltke Sørensen, Grazielle Náthia-Neves, Charlotte Jacobsen

**Affiliations:** National Food Institute, Technical University of Denmark, 2800 Kongens Lyngby, Denmark; saghel@food.dtu.dk (S.G.); adms@food.dtu.dk (A.-D.M.S.); gnev@food.dtu.dk (G.N.-N.)

**Keywords:** *Palmaria palmata*, aqueous extraction, enzymatic hydrolysis, protein, antioxidant activity, rhodophyceae, iron chelation, radical scavenging

## Abstract

This study employed a diverse approach to extract antioxidant peptides from red seaweed *Palmaria palmata*, recognized for its comparatively high protein content. Initially, an aqueous extraction of the entire seaweed was performed, followed by enzymatic hydrolysis of the solid residues prepared from the first step. The effects of three different pH levels (3, 6, and 9) during the aqueous extraction were also examined. Results indicated that the solid fraction from the sequential extraction process contained significantly higher levels of proteins and amino acids than other fractions (*p* < 0.05). Furthermore, the solid fractions (IC_50_ ranging from 2.29 to 8.15 mg.mL^−1^) demonstrated significantly greater free radical scavengers than the liquid fractions (IC_50_ ranging from 9.03 to 10.41 mg.mL^−1^ or not obtained at the highest concentration tested) at both stages of extraction (*p* < 0.05). Among the solid fractions, those produced fractions under alkaline conditions were less effective in radical scavenging than the produced fractions under acidic or neutral conditions. The fractions with most effective metal ion chelating activity were the solid fractions from the enzymatic stage, particularly at pH 3 (IC_50_ = 0.63 ± 0.04 mg.mL^−1^) and pH 6 (IC_50_ = 0.89 ± 0.07 mg.mL^−1^), which were significantly more effective than those from the initial extraction stage (*p* < 0.05). Despite no significant difference in the total phenolic content between these solid fractions and their corresponding liquid fractions (3.79 ± 0.05 vs. 3.48 ± 0.02 mg.mL^−1^ at pH 3 and 2.43 ± 0.22 vs. 2.51 ± 0.00 mg.mL^−1^ at pH 6) (*p* > 0.05), the observed antioxidant properties may be attributed to bioactive amino acids such as histidine, glutamic acid, aspartic acid, tyrosine, and methionine, either as free amino acids or within proteins and peptides.

## 1. Introduction

Seaweeds have emerged as promising marine resources for bioactive compounds with nutritional and medicinal properties [1]. These marine organisms are consumed as staple foods in many regions worldwide and are considered essential components of a healthy diet [2]. Seaweeds contain structurally diverse compounds with various bioactivities, including antioxidant [3,4], antihypertensive [5,6], anti-inflammatory [4], and anticarcinogenic [7] effects. These fast-growing and renewable resources are being explored for novel and sustainable compounds in pharmaceutical, nutraceutical, and cosmetic applications. Notably, bioactive peptides derived from seaweed have gained recognition for their therapeutic potential, showcasing properties such as anti-tumor and blood pressure regulation [8] and antioxidant activity, especially from red algae species such as *Eucheuma cottonii*, *Gracilariopsis lemaneiformis*, *P. palmata*, *Porphyra columbina*, and *Porphyra dioica* [9]. Integrating seaweeds into diets and pharmaceutical formulations holds significant promise in bolstering overall health and combating prevalent health challenges. Furthermore, seaweed is an excellent choice for vegans and vegetarians due to its rich nutrient profile and provides a sustainable and plant-based alternative to traditional protein sources [10].

Although seaweeds have gained attention for the health-promoting effects of their bioactive compounds, such as proteins and polyphenols, their rigid cell walls pose challenges during extraction [11]. To overcome this, various techniques have been studied, including the use of solvents [12], subcritical and supercritical fluids [13], pressurized liquid [14], ultrasound [15,16], microwave [17], and enzymes [18]. Additionally, combinations of these techniques, such as enzymatic/alkaline extraction [11,19], have been explored. One critical underlying factor in extracting bioactive compounds from seaweeds is pH. Manipulating pH can alter the ionic interactions within the cell wall matrix, affecting its permeability and, thus, the release of bioactive compounds [20]. For instance, hydrogen bonds between protein molecules break in highly alkaline environments, increasing the proteins’ surface charge and enhancing their solubility [21]. Conversely, protein aggregation and precipitation occur at the protein’s isoelectric point due to the alteration of surface hydration and electric double layers [21]. Therefore, careful consideration of pH is essential to optimize the extraction efficiency and preserve the integrity of bioactive compounds from seaweeds.

*P. palmata*, a commonly found species of red seaweed, stands out for its relatively high protein content, distinguishing it as a notable source of plant-based protein [22]. The protein content in *P. palmata* is of high quality, meeting human requirements for essential amino acids, and most of these essential amino acids remain consistently present regardless of seasonal variations [23]. Apart from proteins, this species also serves as a valuable source of other nutrients, including phenolic compounds [11]. In this study, we employ a pH-dependent sequential approach to obtain antioxidant peptides from *P. palmata*. We hypothesize that the pH conditions will influence the protein concentration in the solid fractions, making them more suitable substrates for enzymatic hydrolysis. We also expect pH to impact the release of phenolic compounds and their interactions with other compounds, such as proteins and peptides, ultimately defining the properties of the extracts. Therefore, we analyzed the properties of the liquid and solid fractions obtained after aqueous extraction under varying pH conditions, i.e., 3, 6, and 9 (for ease, hereafter referred to as LA3, LA6, and LA9 for liquid fractions and SA3, SA6, and SA9 for solid fractions, respectively). Additionally, our investigation extends to analyzing the properties of the liquid and solid fractions after enzymatic hydrolysis using the solid fractions from the initial aqueous extraction round as substrates (hereafter referred to as LE3, LE6, and LE9 for liquid fractions and SE3, SE6, and SE9 for solid fractions, respectively).

## 2. Results

### 2.1. Protein Content, Protein Recovery, and Degree of Hydrolysis (DH)

**Figure 1:**
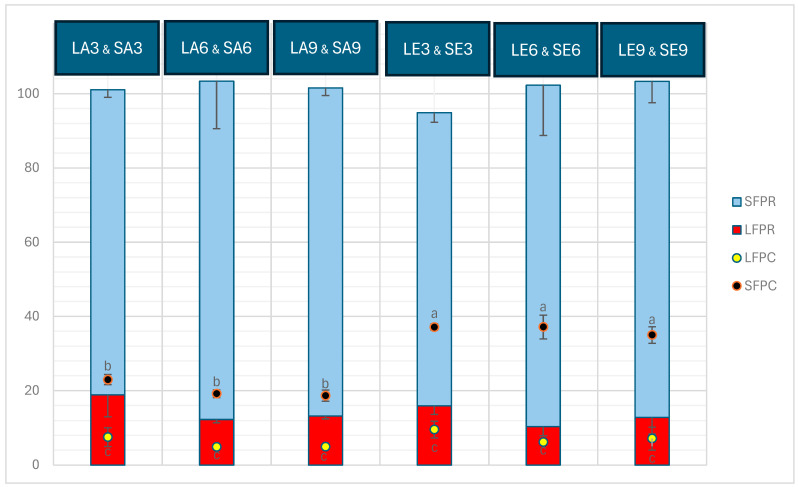
Protein content (%, dry weight) in liquid and solid fractions (LFPC and SFPC, respectively) and protein recovered (%) in liquid and solid fractions (LFPR and SFPR, respectively) after aqueous and enzymatic extraction from *P. palmata*. Data were expressed as mean ± standard deviation. LA and SA denote liquid and solid fractions after aqueous extraction; LE and SE stand for liquid and solid fractions after enzymatic extraction (using the solid fraction from the aqueous stage as substrate), respectively; the numbers show the pH values tested. The letters ‘a’, ‘b’, and ‘c’ denote significant differences among the treatments in terms of protein content (*p* < 0.05). Within neither the liquid nor the solid fraction, the samples exhibit statistically significant differences in protein recovery (*p* > 0.05).

The freeze-dried seaweed used as the substrate for the aqueous extraction in this study contained 13.46 ± 0.02% protein. The protein content (%, dry weight) and protein recovery in the liquid and solid fractions obtained after aqueous extraction on seaweed and the subsequent enzymatic extraction on the solid fraction obtained from the aqueous extraction are depicted in Figure 1. The protein content in solid fractions significantly outweighed that of liquid fractions (*p* < 0.05). The solid fractions obtained after enzymatic extraction (SE3, SE6, and SE9) contained considerable concentrations of protein, which were significantly higher than those of SA3, SA6, and SA9 obtained before enzymatic extraction (*p* < 0.05). However, within the SA and SE groups, there were no significant differences between the three pH levels (*p* > 0.05). The protein content in the liquid fractions ranged from approximately 5% to 9.5%, with no significant difference observed among or within the LA and LE samples (*p* > 0.05). Furthermore, protein recovered in liquid and solid fractions was circa 10–19% and 79–92%, respectively. Protein recovery in the liquid fractions obtained at pH 3 (LA3 and LE3) was higher than that in those fractions at pH 6 (LA6 and LE6) and pH 9 (LA9 and LE9), whereas a reverse trend is noted in protein recovery in the solid fractions at pH 3 (SA3 and SE3) compared with that at pH 6 (SA6 and SE6) and pH 9 (SA9 and SE9). SE samples exhibited the highest protein recovery across all three pH levels tested, surpassing the protein recovery in SA samples, which was higher than in the liquid fractions (LA and LE). No significant differences were observed within the SE samples at different pH levels, nor within the SA samples at different pH levels (*p* > 0.05). Additionally, there were no significant differences among all the LA and LE samples at any of the pH levels tested (*p* > 0.05).

**Figure 2:**
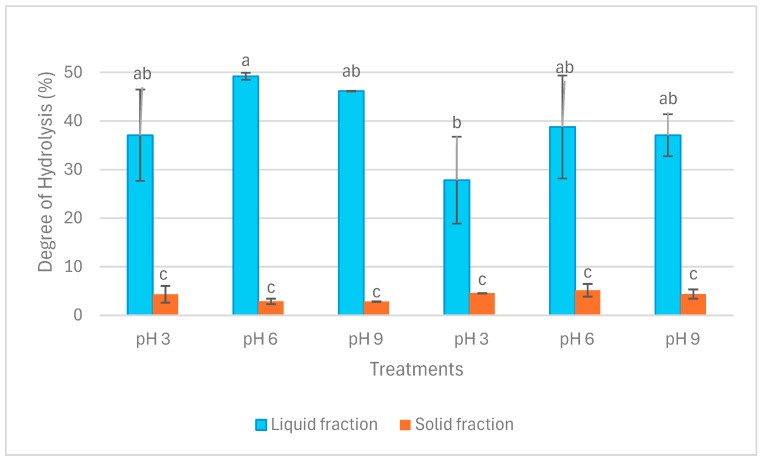
Degree of hydrolysis (DH) in liquid and solid fractions after aqueous and enzymatic extraction from *P. palmata*. Data were expressed as mean ± standard deviation. LA and SA denote liquid and solid fractions after aqueous extraction, respectively; LE and SE stand for liquid and solid fractions after enzymatic extraction (using the solid fraction from aqueous stage as substrate), respectively. The different letters (‘a’, ‘b’, and ‘c’) denote significant differences among the treatments in terms of DH (*p* < 0.05).

Figure 2 illustrates the results of the degree of hydrolysis (DH) in liquid and solid fractions. DH in liquid fractions after both extraction operations was significantly higher than in solid fractions (*p* < 0.05). Within the liquid fractions, DH ranged from circa 30% in LE3 to 50% in LA6. Nevertheless, DH in solid fractions ranged between approximately 3% and 5%. The difference between LE3 and LA6 in terms of DH was significant (*p* < 0.05), whereas no other significant difference was detected in DH among the liquid fractions (*p* > 0.05). In addition, there was no significant difference in terms of DH among the solid fractions (*p* > 0.05).

### 2.2. Amino Acid Composition

The amino acid profiles of LA, SA, LE, and SE samples at pH 3, 6, and 9 are presented in Table 1. Overall, a general trend can be seen in the amino acid content of the samples: SE > SA > LA or LE. The amino acid contents of SE samples at all three pH conditions were significantly higher than those of the other samples (*p* < 0.05). No significant difference was observed among SE3, 6, and 9 in terms of amino acid content (*p* > 0.05), except for arginine, which was significantly lower in SE9 compared to SE3 and SE6 (*p* < 0.05). A similar trend was observed for SA samples (*p* > 0.05) with some exceptions; for instance, arginine, histidine, glutamic acid, and aspartic acid were significantly higher in SA3 than in SA9 (*p* < 0.05). LA and LE samples’ noticeably lower amino acid contents at all three pH values corresponded well with their protein contents (see Section 2.1). Except for glutamic acid (*p* < 0.05), no significant differences were detected among the liquid fractions from both extraction steps (*p* > 0.05). Furthermore, histidine was absent in the liquid fractions but present in the solid samples. Additionally, cystine was only detected in SE samples, and its concentration was significantly higher in SE3 compared to SE6 and SE9 (*p* < 0.05). It should be noted that tryptophan (destroyed during acid hydrolysis), cysteine (if present, converted to cystine), and lysine (not quantified) were absent in the profiles. The sum of essential amino acids and essential/non-essential amino acids ratio were significantly higher in SE than in SA samples and higher in SA samples compared to LA and LE samples (*p* < 0.05). There was no significant effect of pH on this ratio (*p* > 0.05).

### 2.3. Total Phenolic Content

**Figure 3:**
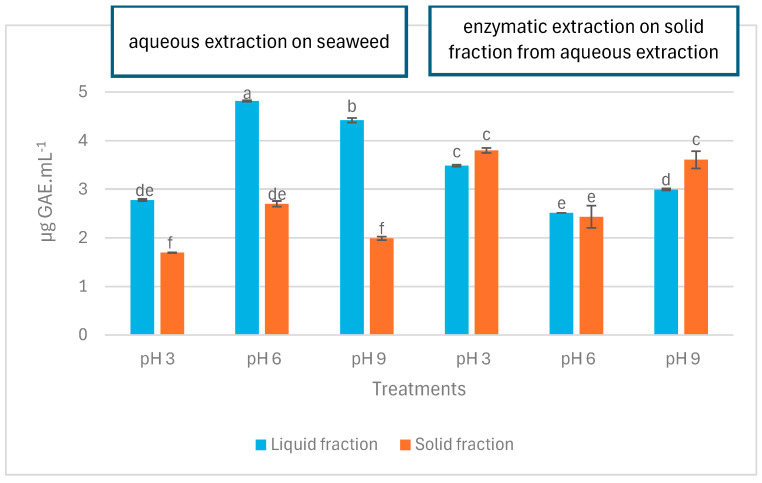
Total phenolic content (TPC) in liquid and solid fractions after aqueous and enzymatic extraction from *P. palmata*. Data were expressed as mean ± standard deviation. LA (first three blue bars) and SA (first three orange bars) denote liquid and solid fractions after aqueous extraction, respectively; LE (second three blue bars) and SE (second three orange bars) stand for liquid and solid fractions after enzymatic extraction (using the solid fraction from aqueous stage as substrate), respectively. Different letters denote significant differences among the treatments in terms of TPC (*p* < 0.05).

The total phenolic content (TPC) of LA, SA, LE, and SE samples at pH 3, 6, and 9 are depicted in Figure 3. At first glance, it is observed that the LA samples contained significantly more phenolic compounds compared to the SA samples (*p* < 0.05). In contrast, TPC in LE showed no significant difference (*p* > 0.05) or was significantly lower compared to SE (*p* < 0.05). At all pH levels tested, TPC in LA was significantly higher than in SA (*p* < 0.05). However, different results were observed for LE and SE samples regarding the effect of pH on TPC. At pH 3 and pH 6, there was no significant difference between LE and SE (*p* > 0.05), and at pH 9, TPC in LE was significantly lower than SE (*p* < 0.05).

### 2.4. Antioxidant Properties

Table 2 presents the IC_50_ values for 1,1-diphenyl-2-picrylhydrazyl (DPPH) radical scavenging activity and Fe^2+^ chelating activity of LA, SA, LE, and SE samples at pH 3, 6, and 9. The highest radical scavenging activities were observed for SA and SE samples, especially at pH 3 (IC_50_ = 3.97 ± 0.19 mg.mL^−1^ for SA3 and IC_50_ = 2.29 ± 1.00 mg.mL^−1^ for SE3) and pH 6 (IC_50_ = 2.85 ± 0.08 mg.mL^−1^ for SA6 and IC_50_ = 2.92 ± 0.02 mg.mL^−1^ for SE6). No significant difference was witnessed between SE3 and SE6 and their SA counterparts (*p* > 0.05). The radical scavenging activity of SA9 was significantly lower than other solid fractions (*p* < 0.05), except for SE9 (*p* > 0.05). The liquid fractions at pH 9, whether after aqueous or subsequent enzymatic treatments, did not scavenge 50% of free radicals at the maximum concentration of extract tested (i.e., 16 mg.mL^−1^). The IC_50_ values of LA and LE at pH 3 and pH 6 ranged between circa 9 and 10.5 mg.mL^−1^ (*p* > 0.05).

The metal ion chelating activity of SE samples was significantly higher across all tested pH levels compared to SA samples (IC_50_ ranging from 0.63 to 1.35 mg.mL^−1^ for the former as opposed to 4.81–11.84 mg.mL^−1^ for the latter) (*p* < 0.05). However, there were no significant differences among SE samples regarding Fe^2+^ chelating activity (*p* > 0.05). In addition, the chelating activity of SA3 was significantly higher than that of SE6 and SE9 (*p* < 0.05). Among the liquid fractions, LA9 exhibited the highest chelating activity (IC_50_ = 1.26 ± 0.07 mg.mL^−1^), significantly surpassing all other LA and LE samples (*p* < 0.05). Even at the highest concentration tested, 16 mg.mL^−1^, LA3 could not chelate at least 50% of the Fe^2+^ ions. SE3 demonstrated the most potent Fe^2+^ ion chelating capability of all the samples evaluated, achieving 50% chelation of the ions at a mere concentration of 0.63 mg.mL^−1^.

## 3. Discussion

### 3.1. Protein Content, Protein Recovery, and Degree of Hydrolysis

Since the results obtained in our previous study showed the very high solubility of proteins at alkaline pH after enzymatic/alkaline extraction of proteins from *P. palmata* (>90% protein recovery in liquid fractions vs. <5% protein recovery in solid fractions) [11], it was expected that the application of alkaline (or acidic) pH in this study would also contribute to the release and solubility of protein in the liquid fraction. The logic behind this hypothesis was that any deviation from the isoelectric point of proteins, whether toward alkaline or acidic conditions, could solubilize proteins in liquid fractions [20]. At pH values away from the isoelectric point, the surface charges of proteins tend to be either negative at alkaline conditions or positive at acidic conditions, which results in the weakened hydrophobic interaction and stronger electrostatic repulsion between proteins contributing to the interaction between protein and water and therefore increased protein solubility [24]. However, the results of this study revealed that neither alkaline nor acidic pH could contribute to the solubilization of proteins in the supernatant during aqueous extraction, as evidenced by substantially higher protein concentration and protein recovery in solid fractions after the extraction at varying pH values. One potential reason for this observation might be the presence of polysaccharides in seaweed. These polysaccharides are crucial components of seaweed cell walls and have strong interactions with bioactive compounds such as proteins [25]. Although alkaline extraction coupled with enzymatic pretreatment in previous studies yielded higher protein contents in liquid fractions [11,19], alkaline or acidic pH during aqueous extraction cannot contribute to the solubilization of proteins from red seaweed. This could be partly due to the heterogeneous nature of seaweed proteins that necessitates the application of more than one extraction method to disintegrate seaweed cell walls and meet the solubility requirements of proteins [26].

Another explanation for low protein solubility after aqueous extraction at different pH values is the possibility of interactions between proteins and polysaccharides after extraction. Once proteins and peptides are released into the solution, they can reassociate with polysaccharides in the extract, forming insoluble complexes. In the solution, hydrogen bonds and electrostatic interactions between proteins and polysaccharides can form stable complexes [27]. When proteins and polysaccharides interact in a solution, they can form complexes through a process known as coacervation. Coacervation involves the associative phase separation between proteins and polysaccharides, forming two distinct phases: a biopolymer-rich phase and a biopolymer-poor phase. The biopolymer-rich phase can exhibit different states, including liquid coacervates and solid precipitates [28]. Depending on the concentration and types of proteins and polysaccharides, as well as the pH and ionic strength of the solution, these complexes can precipitate out of the solution, contributing to the protein content in the solid fraction [29]. At certain pH levels, the charges might promote binding and complex formation. For example, at low or high pH conditions, depending on their isoelectric point, proteins might have a net positive or negative charge that can interact strongly with charged polysaccharides [30]. However, these effects of pH in the current study were outweighed by other factors since no significant differences were observed in the solubility or precipitation of proteins at varying pH conditions (*p* > 0.05). It was highlighted that besides pH, other factors, such as polysaccharide type, ionic strength, temperature, etc., could also determine the solubility of proteins within the complexes [31].

Furthermore, the presence of salts can shield electrostatic interactions, potentially reducing the formation of complexes. However, these interactions might be stronger at low ionic strengths, promoting complexation [32]. Therefore, the presence of naturally occurring salts in *P. palmata* can influence the electrostatic interactions between proteins and polysaccharides, impacting the formation and stability of insoluble complexes. The seaweed naturally contains a significant amount of minerals, including sodium, potassium, magnesium, and calcium [22], which can contribute to the overall ionic strength of the seaweed matrix. In this regard, one may consider the protein–polysaccharide complex in the current study as polyelectrolyte multilayers that are formed by the layer-by-layer disposition of oppositely charged polyelectrolytes (e.g., proteins and polysaccharides) and are affected by the inherent salt content of the seaweed. However, this proposition should be taken with care because (i) proteins generally do not possess the flexibility and geometry required to be included in polyelectrolyte–polyelectrolyte systems; (ii) in protein–polyelectrolyte systems, pH modulation primarily impacts protein charge, while ionic strength plays a more complex role, increasing polyelectrolyte’s configurational entropy and affecting the entropy of small ion release; and (iii) when replacing a polyelectrolyte with a protein in the system, the role of configurational entropy is significantly diminished [29]. Two other factors that could be envisaged as drivers of observed protein dissolubility in the current study regarding the formation of protein–polysaccharide complexes are temperature and polysaccharide type and concentration. Higher temperatures can increase the solubility of proteins and kinetic energy, potentially promoting interactions between proteins and polysaccharides and the formation of insoluble complexes. Nevertheless, temperature rise below the protein’s denaturation point was reported to decrease the interaction strength between proteins and polysaccharides during complex coacervation [29]. The temperature applied in the current study for the aqueous extraction was 50 °C, below most proteins’ denaturation threshold. Therefore, one should think twice before accrediting the role of extraction temperature in the present research on the precipitation of a large proportion of proteins in solid fractions.

A high concentration of polysaccharides can enhance the likelihood of interactions and complex formation with proteins. These polysaccharides can cause structural rearrangements in proteins, forming insoluble aggregates [33]. Carbohydrates comprise up to 74% of *P. palmata*’s dry weight, with xylans being the primary component of its cell walls. These xylans consist mainly of β-(1→4)- and β-(1→3)-linked D-xylose units and are largely insoluble. Additionally, minor amounts of cellulose (around 3% dry weight), an insoluble glucan, are present as structural carbohydrates. *P. palmata* also contains water-soluble, low molecular weight carbohydrates, primarily floridoside (α-D-galactopyranosyl-(1–2)-glycerol) and smaller amounts of floridean starch. The floridoside content varies seasonally, ranging from less than 5% (dry weight) in winter to up to 25% in summer [22]. This aligns with the results of the present study, as the biomass used for peptide extraction was harvested in winter when the seaweed contains more insoluble polysaccharides.

We anticipated that the subsequent enzymatic extraction, utilizing the solid fractions obtained from the initial aqueous extraction as substrates, would solubilize proteins. Consequently, we expected the resulting liquid fractions to have significantly higher protein content than LAs. However, our expectations were not met, and once again, most proteins precipitated in the solid fractions. The significant protein precipitation observed in solid fractions after both aqueous and enzymatic extraction stages suggests that the proteins are strongly interacting with other components (possibly polysaccharides) or aggregated in a manner that resists solubilization under the extraction conditions used. One possible explanation for this finding is that the enzymatic hydrolysis with Flavourzyme^®^ has broken down proteins into peptides of various lengths. These peptides can have exposed amino and carboxyl groups, which can interact with hydroxyl and carbonyl groups present in seaweed polysaccharides [34]. Future studies are directed toward scrutinizing whether such a conjugation could happen in the presence of seaweed polysaccharides and hydrolyzed proteins. Under certain conditions, especially in complex biological matrices like seaweed extracts, (non-covalent) interactions between hydrolyzed peptides and seaweed carbohydrates can form complexes or conjugates. This presents an important avenue for future research.

The modest DH observed in the solid fractions may reflect that the proteins present have been subject to limited hydrolysis. This could imply either a natural resistance of the proteins in the solid fractions to enzymatic degradation or that the hydrolysis conditions were not conducive to a more complete breakdown of these proteins. It could also imply that these proteins form aggregates or complexes that protect them from enzymatic action. Studies have shown that polysaccharides like xylan interact with proteins and proteolytic enzymes, leading to reduced protein hydrolysis in seaweed [35]. Notably, the combinational or sequential use of polysaccharidase alongside the protease in our procedure to extract peptides from the seaweed merits further investigation. In addition, the high DH in liquid fractions indicates that the proteins present in these fractions were extensively hydrolyzed. The DH values observed in the present study for the liquid fractions after the initial aqueous and subsequent enzymatic extractions are higher than those reported in our previously published paper [11] following enzymatic/alkaline extraction. However, these DH values are comparable to those observed in our ongoing work (currently under preparation for publication) after enzymatic treatment without the subsequent alkaline extraction stage. This discrepancy could be because alkaline conditions can denature proteins, potentially exposing more peptide bonds initially while also causing changes in protein conformation [36] that render some bonds less accessible to enzymes or the OPA (o-phthaldialdehyde) reagent used for DH measurement. The OPA method is a spectrophotometric assay that relies on the chemical reaction between OPA and primary amines in the presence of a thiol (such as dithiothreitol, DTT) to form a highly fluorescent isoindole derivative [37,38]. The fluorescence intensity is directly proportional to the concentration of free primary amines in the sample, which corresponds to the extent of protein hydrolysis. Alkaline extraction can cause protein denaturation, which involves unfolding protein structures. This can expose hydrophobic regions, forming (still soluble) aggregates [39]. These aggregates might bury free amino groups within their structure, making them less reactive. Alkaline extraction can also induce chemical modifications such as deamidation of aspartic acid and glutamic acid [40], which can alter the availability and reactivity of amino groups, leading to lower DH values measured by the OPA method. Furthermore, enzymes might be susceptible to strongly alkaline conditions [41]. Consequently, any residual enzymatic activity might be lost during the alkaline extraction step, halting further hydrolysis that could have occurred if conditions were maintained for enzyme activity. It should be noted that enzymatic hydrolysis in weakly buffered systems in this study may result in greater fluctuations in pH during the reaction, which can affect enzyme activity and stability. Furthermore, higher enzyme concentration may yield different results, which necessitates the optimization of enzyme concentration. This can be achieved through systematic experimentation to determine the optimal balance between enzyme efficiency and cost-effectiveness. Such optimization requires further research.

### 3.2. Amino Acid Composition

The general trend observed for the total amino acid composition of liquid and solid fractions corresponded well with this study’s findings regarding the samples’ protein content based on the dry matter. Therefore, readers are referred to the discussion in the previous section for clarifications and interpretations on the significant differences between solid fractions from two extraction stages and between solid and liquid fractions regarding amino acid profiles. However, individual differences observed within each group of the samples are interpreted here.

The first notable observation was attributed to the lower arginine content in SE9 compared to SE3 and SE6. One plausible explanation involves the distinct behavior of arginine counterions under alkaline conditions, leading to the dissociation of arginine from tightly bound micellar aggregates. Consequently, more arginine may diffuse into the soluble fraction, resulting in a lower observed amount in the solid fraction. This trend is reflected by the slightly higher (though not statistically significant) arginine content in LE9 compared to LE3 and LE6 (Table 1). At alkaline pH, arginine tends to adopt a zwitterionic state rather than maintaining a net positive charge. This change in charge state can trigger the dissociation of arginine molecules from micellar structures, particularly as the primary amine groups deprotonate. This dissociation process becomes more pronounced at higher pH levels, approaching complete dissociation [42]. One should also consider the effect of pH on protease activity, which could potentially promote secondary reactions that degrade arginine. However, this hypothesis seems less likely because a similar trend was observed in liquid and solid fractions obtained after the initial aqueous extraction, where no enzymatic treatment was applied. Moreover, in addition to arginine, our results denoted that histidine, glutamic acid, and aspartic acid were significantly higher in SA3 than in SA9 (*p* < 0.05). Since arginine and histidine are both basic amino acids, the explanation mentioned above regarding the dissociation of the amino acids from micellar aggregates and diffusion into soluble fractions may also be the case for histidine. However, amino acids with acidic side chains like aspartic acid may undergo chemical modifications such as isomerization and racemization under different pH conditions [43], which can affect the detectable content of these amino acids during analysis.

In addition, histidine was not found in the liquid fractions but in the solid samples. This observation may be attributed to the distinctive structural feature of histidine, which includes a basic imidazole group on its side chain [44]. This characteristic could facilitate robust complexation with macromolecules in the seaweed extract matrix, such as polyphenols and polysaccharides. Consequently, this interaction may result in histidine predominantly residing in the solid fractions, rendering it undetectable in the liquid fractions. Furthermore, the imidazole group of histidine facilitates molecular interactions (cation-π, π-π stacking, hydrogen-π, coordinate bond, and hydrogen bond interactions) with other amino acids [45], which might lead to the formation of insoluble complexes that aggregate in the solid fractions. In addition, cystine was detected exclusively in the SE samples, with its concentration being significantly higher in SE3 compared to SE6 and SE9. The emergence of cystine in the solid fractions following the enzymatic process, in contrast to its non-detection in the solids post-aqueous extraction, indicates that enzymatic hydrolysis was essential in liberating cystine from its formerly attached state. This implies that the pH conditions applied during the aqueous phase were not adequately potent to dissociate cystine from its native structure or the complexes in which it may have been trapped.

### 3.3. Total Phenolic Content

The noticeably higher total phenolic content in the liquid fractions compared to the solid fractions after aqueous extraction can be attributed to the inherent solubility characteristics of phenolic compounds. Phenolics are more soluble in water and other solvents compared to being bound to solid matrices. During extraction, the solvent can penetrate the substrate and solubilize the phenolic compounds, which are subsequently preserved in the liquid phase [46]. Phenolic compounds are sensitive to pH changes due to their chemical structure. As expected, the solubility of phenolic compounds after the aqueous extraction was significantly higher at pH 9 compared to pH 3. The alkaline conditions can deprotonate phenolic hydroxyl groups [47], making them more soluble in water. In contrast, acidic conditions can lead to the protonation of phenolic compounds [48], making them less soluble and more likely to bind to solid particles, resulting in lower extraction into the liquid fraction. Surprisingly, the solubility of phenolic compounds after aqueous extraction was higher at pH 6 than under alkaline conditions. This is probably due to the varying susceptibility of phenolic compounds with different structures to pH [49]. Interestingly, an opposite trend was observed in the solubility of phenolic compounds after the subsequent enzymatic treatment, where the highest and lowest TPC were observed in acidic and almost neutral conditions, respectively. This could be due to the structure and accessibility of the protein–phenolic complexes in the residue as influenced by pH. In acidic or alkaline conditions, proteins might be more unfolded and accessible to protease, facilitating better breakdown and release of phenolics into the solvent. In contrast, phenolics might form stronger or more stable complexes with proteins at near-neutral pH, reducing solubility and measured phenolic compounds. This is in line with a study that reported the highest rate of protein–polyphenol (β-lactoglobulin-caffeic acid) conjugation occurred at pH 6 [50].

### 3.4. Antioxidant Properties

The solid fractions exhibited greater free radical scavenging capabilities in both extraction phases than their liquid counterparts. This finding diverges from our TPC results, where liquid fractions typically had higher levels of phenolic compounds. This observation contrasts earlier studies that emphasized the significant contribution of phenolic compounds to the radical scavenging efficacy of seaweed extracts [51]. However, the current study suggests that proteins, particularly peptides, free amino acids, and/or their complexes with other macromolecules like polyphenols or carbohydrates, may play a leading role in neutralizing free radicals in seaweed products. The substantial protein concentration in the solid fractions, noted after the initial aqueous extraction and the subsequent enzymatic treatment, supports this hypothesis, marking a stark difference from the protein levels in the liquid fractions. Our results also indicated that the enzymatic hydrolysis of solid residues from the initial aqueous extraction yielded extracts with slightly better radical scavenging properties. This corroborates prior research highlighting the significance of protease treatment in producing smaller peptides and improved antioxidant effects [9]. However, this conclusion should be approached with prudence due to the low DH observed in the solid fractions. In addition, considering the negligible variance among the solid fractions obtained from various extraction phases at each tested pH level, the practicality of applying additional enzymatic processing should be contemplated, particularly when aiming to obtain fractions with strong free radical neutralizing abilities.

It is important to consider the connection between the DPPH radical scavenging activity observed in the samples of this study and their amino acid profiles. The lack of histidine in the liquid fractions, contrasted with its presence in the solid fractions, suggests that histidine may play a significant role in neutralizing free radicals. The efficiency of histidine in scavenging DPPH radicals is likely attributed to its imidazole ring structure [52]. The present study highlights the importance of histidine in the free radical scavenging capabilities of the examined fractions. The notable variance in histidine levels across the samples correlates with the observed differences in DPPH radical scavenging activity. Specifically, SA9 exhibited a markedly lower DPPH radical scavenging activity than its counterparts at the other two pH values tested. This coincides with its reduced histidine content, especially when compared to SA3, where the disparity was significant (*p* < 0.05). A comparable pattern was noted in SE samples, where a decrease in histidine was associated with diminished free radical scavenging activity. This observation extends to glutamic acid and aspartic acid, suggesting their potential involvement in the DPPH radical scavenging process of the solid fractions. Research indicates that sequences of electron-donating units like glutamic acid and aspartic acid within peptide chains enhance the neutralization of free radicals [53]. Tyrosine may also contribute to the notably greater free radical scavenging capabilities of solid residues relative to liquid fractions, as it is found in higher concentrations within the solids. The presence of a hydroxyl group in tyrosine has been identified as a key factor in its effectiveness as an antioxidant amino acid [54]. The involvement of amino acids in antioxidant activities was also highlighted in studies examining the bioactivity of peptides from seaweed [9,11] and other aquatic sources, including marine by-products [54,55,56] and crustaceans [57].

Regarding the metal ion chelating properties of the samples, LA3 was ineffective in chelating Fe^2+^, while SA3 showed moderate activity. This could be due to the protonation of functional groups at low pH, reducing the availability of chelating agents in the liquid fraction. In contrast, in the fraction obtained at alkaline pH, the chelating activity of LA was significantly enhanced, suggesting that higher pH levels favor the solubilization of chelating agents into the liquid fraction. This phenomenon might be partially attributed to the substantial phenolic content in LA9. However, there must be other contributing factors because if phenolic content were the sole determinant, then LA6, which had a higher phenolic content than LA9, would be expected to exhibit greater chelating activity, yet it did not. This variation may stem from the distinct structural configurations of the phenolic compounds and the differing dynamics of complex formation and stability [58]. An alternative explanation for LA9’s enhanced Fe^2+^ chelating capability might be that an alkaline environment promotes the release of polysaccharides [59]. It has been noted that polysaccharides found in red seaweed are effective at chelating metal ions [60]. Consequently, the increased release of these polysaccharides in an alkaline setting could contribute to the greater metal chelating efficiency of LA9, which could occur either through the direct action of these chelating polysaccharides or by fostering the creation of more effective chelating complexes with proteins and/or polyphenols.

Enzymatic hydrolysis significantly enhanced the metal chelating capacity of the solid fractions across all pH levels, with particularly notable results at a pH of 3 and a pH of 6. This improvement is likely due to the breakdown of proteins by the protease applied, releasing peptides and amino acids with strong metal-chelating properties. This is particularly noteworthy considering that the total phenolic contents of SE3 and SE6 did not differ significantly from their LE counterparts (*p* > 0.05), which suggests that the peptides and amino acids play a more dominant role in chelating metals in the solid fraction. The metal chelating capacity has been linked to the size of peptides, indicating that multiple negatively charged groups may improve the binding with metal ions [53]. Thus, it is reasonable to deduce that the high Fe^2+^ chelating attributes of the SE samples in this study are due to the shorter peptides generated by the protease’s action on the whole or partially broken-down proteins in the solid fractions obtained from the initial aqueous extraction. The elevated chelating activity observed in SE samples may be due, in part, to the notably greater levels of methionine and histidine they contain relative to other samples (Table 1). Methionine [61] and histidine [62] are recognized for their effective metal ion chelation. As such, its inclusion in peptides can greatly enhance the total metal chelating capacity of the samples. Additionally, the presence of cystine, cysteine’s dimeric variant, exclusively in SE samples might play a role in their pronounced chelating capacity. Nevertheless, caution is advised when concluding this since cystine’s chelating characteristics may vary from those of cysteine, which is recognized for its strong metal ion chelating properties [61]. Furthermore, the chelating activity exhibited a steady increase as the pH level was reduced from 9 to 3 in SE samples, although this increase did not present a significant difference (*p* > 0.05). Like the outcomes of free radical scavenging, the modest reduction in histidine levels at elevated pH could account for the slightly diminished chelating activity noted in SE samples with a pH of 9.

## 4. Materials and Methods

### 4.1. Seaweed Biomass Preparation

Air-dried *P. palmata* from a batch harvested between late autumn and early winter in 2023 from Faroe Islands coasts was purchased from a Danish company (DanskTANG, Nykøbing Sj., Denmark). To decide on the feasibility of freeze-drying the biomass before extraction, the dry matter of the retained biomass was calculated after vaporization at 102–105 °C for 24 h, and the dry matter content was expressed as % of the biomass weight. The biomass was freeze-dried using a ScanVac CoolSafe freeze-dryer (LaboGene A/S, Allerød, Denmark) to remove as much moisture as possible. The freeze-dried seaweed biomass was then pulverized to approximately 0.5–1.0 cm particle size using a laboratory mill (KN 295 Knifetec™, Foss A/S, Hillerød, Denmark). Afterward, the resulting powder was stored in zip-lock plastic bags at −20 °C in dark conditions.

### 4.2. Enzymes and Chemicals

Flavourzyme^®^, 500–1000 leucine aminopeptidase units (LAPU).g^−1^, was kindly provided by Novenesis A/S (formerly known as Novozymes A/S) (Bagsværd, Denmark). All solvents used were of high-performance liquid chromatography (HPLC) grade and purchased from Lab-Scan (Dublin, Ireland). Amino acid standards (arginine, serine, glycine, threonine, alanine, proline, valine, methionine, aspartic acid, histidine, lysine, glutamic acid, leucine, phenylalanine, isoleucine, cystine, and tyrosine) were purchased from Sigma–Aldrich (St. Louis, MO, USA). HPLC-grade water was prepared at DTU Food using a Milli-Q^®^ Advantage A10 water deionizing system from Millipore Corporation (Billerica, MA, USA). Butylated hydroxytoluene (BHT), ethylenediaminetetraacetic acid (EDTA), and DPPH radical were obtained from Sigma–Aldrich (Steinheim, Germany). All other chemicals were obtained from Merck (Darmstadt, Germany).

### 4.3. Aqueous Extraction 

To determine the effect of pH on the properties of liquid and solid fractions obtained after aqueous extraction, six blue-capped bottles (treatments in duplicate) containing 5 g of biomass powder and 100 mL of deionized water (1:20 *w*/*v*) were placed in a water bath at 50 °C for 1 h for biomass rehydration. Afterward, the pH values for each treatment were adjusted to 3, 6, and 9 using either 1.0 M hydrochloric acid (HCl) or 1.0 M sodium carbonate (Na_2_CO_3_). Sodium carbonate was claimed to facilitate extraction by opening the seaweed structure [19]. The aqueous extraction was performed in a shaking water bath at 80 rpm and 50 °C for 14 h [19]. The pH values of the samples remained relatively stable during the extraction process, with a maximum change of only 0.5 units across all initial pH conditions (3, 6, and 9). Then, the content of each bottle was filtered through a sieve (ca. 1 mm mesh size), and the resulting liquid and solid fractions were pre-frozen at −20 °C for 2 h and then transferred to a −80 °C freezer for 6 h before they were freeze-dried (LaboGene A/S, Allerød, Denmark). The resulting powders were transferred to zip-lock plastic bags and stored at −80 °C until analysis. In the context of mass balance calculations, all fractions were weighed using a laboratory balance with a readability of 0.01 g at different stages.

### 4.4. Enzymatic Hydrolysis

Flavourzyme^®^ at a concentration of 2% protein content was employed to perform enzymatic hydrolysis using the solid fractions from the previous stage as substrates. For this aim, 2 g of freeze-dried solid fractions from the initial aqueous extraction stage were rehydrated with 40 mL of deionized water (1:20 *w*/*v*) in the shaking water bath at 80 rpm and 50 °C for 1 h. Afterward, the pH was adjusted to 7–7.2 using 1.0 M sodium hydroxide (NaOH) (as recommended in [63]) before introducing the enzyme. Enzymatic extraction was performed in the shaking water bath at 80 rpm and 50 °C for 24 h. Afterward, supernatants and solid residues were collected after centrifuging the content of each bottle at 4400 g for 15 min at 4 °C. Then, all fractions were freeze-dried and stored as explained in the initial aqueous stage. Again, the fractions were weighed for mass balance calculation, as explained above.

### 4.5. Protein Content and Recovery

To measure the protein content of biomass powder and freeze-dried fractions, the total nitrogen content of the samples was determined through the Dumas combustion method using a fully automated rapid MAX N (Elementar Analysensysteme GmbH, Langenselbold, Germany). Approximately 200 mg of samples were fed into the system, and the exact weight was recorded. The protein content was determined by multiplying the nitrogen content by a factor of 5.0 [11].

Protein recovery in the samples were calculated based on the following equation:

$$Protein\;recovery\;in\;fraction\;(\%)\;=\;\frac{M_{F}\;\times \;P_{F}}{M_{S}\;\times \;P_{S}}$$

where *M_F_*, *P_F_*, *M_S_*, and *P_S_* stand for the mass of the fraction, the protein percentage of the fraction, the mass of the seaweed, and the protein percentage of the seaweed, respectively.

### 4.6. Degree of Hydrolysis (DH)

The OPA assay was used to assess the DH, following the method described in [64]. Briefly, the OPA reagent was prepared by combining 10 mL of 0.15 M Na₂CO₃•10H₂O, 10 mL of 0.6 M sodium bicarbonate (NaHCO₃), and 88 mg of DTT. Separately, 80 mg of OPA was dissolved in 2 mL of 96% ethanol, then mixed with 10 mL of 1% SDS. This solution was combined with the DTT mixture and diluted to 100 mL with distilled water. Samples were diluted to a protein concentration of 0.05–0.25% and mixed with the OPA reagent in a microplate. Absorbance was measured at 340 nm, and an L-serine calibration curve was used for quantification. The serine equivalent for the samples was determined as outlined below.

$$\mathrm{Sample}\;(\mathrm{mg}\;\mathrm{Ser}.{\mathrm{mL}}^{-1})\;=\;\frac{({\mathrm{Abs}}_{\mathrm{sample}}\;-\;{\mathrm{Abs}}_{\mathrm{blank}})\;-\;\mathrm{intercept}}{\mathrm{slope}}\;\times \;\mathrm{DF}$$

*Abs_sample_*, *Abs_blank_*, and DF denote the sample’s absorbance, the blank’s absorbance, and the dilution factor, respectively. *Intercept* and *slope* are acquired from the L-serine calibration curve. DH (%) is then calculated as shown below.

$$\mathrm{DH}\;(\%)\;=\;\frac{\mathrm{Sample}\;(\mathrm{mg}\;\mathrm{Ser}.{\mathrm{mL}}^{-1})}{P\;\times \;10}\;\times \;100$$

where *P* stands for the protein content in percentage. The measurement of each dilution was carried out in duplicate.

### 4.7. Amino Acid Profile

Approximately 30 mg of the dried sample was hydrolyzed with 6 M HCl at 110 °C for 18 h. Afterward, the hydrolysates were filtered into 4 mL vials through 0.22 µm cellulose acetate spray filters using 1 mL syringes, and then 100 µL of the filtered hydrolysates were pipetted into 4 mL vials. The pH adjustment was carried out by slowly adding 1.5 mL of 0.2 M KOH to the hydrolysates, followed by an additional 1.6 mL of ammonium acetate buffer (100 mM; pH 3.1 adjusted with formic acid) to obtain a dilution factor of 32. The amino acid composition was determined by liquid chromatography using mass spectrometry (Agilent 1260 Infinity II Series, LC/MSD Trap, Agilent technologies, Santa Clara, CA, USA) with a BioZen 2.6 µm Glycan, 100 mm × 2.1 mm (00D-4773-AN) column (Phenomenex, Torrance, CA, USA) connected to a Quadrupole 6120 MS (Agilent Technologies, USA) with an ESI ion source. The following settings were used: a flow rate of 0.5 mL.min^−1^, a column temperature of 40 °C, 1 µL injection volume, and 16 min run time. A gradient mix of two mobile phases, A (10 mM ammonium formate in acetonitrile) and B (10 mM ammonium formate in MilliQ water), was used as follows: 0–2 min 0–5% phase B, 2–7 min 5–20% phase B, 7–8 min 20–80% phase B, 12.1 min 0% phase B, and 12.1–16 min 0% phase B. A mix of amino acid standards containing 17 amino acids (not containing glutamine, tryptophan, or asparagine) was run in five different concentrations to make standard curves. Samples were analyzed, and amino acids were quantitated using MassHunter Quantitative Analysis version 7.0 software. Due to the initial hydrolyzation of the samples, the method can’t detect glutamine, asparagine, tryptophan, or cysteine. Glutamine is hydrolyzed into glutamic acid, while asparagine is hydrolyzed into aspartic acid. Tryptophan and cysteine are destroyed during hydrolysis.

### 4.8. Total Phenolic Content

TPC in the liquid and solid fractions was determined according to [65]. An aliquot (100 μL) of each sample was mixed with 0.75 mL of Folin–Ciocalteu reagent (1:10 diluted) and left at room temperature for 5 min. Sodium bicarbonate (6%, 0.75 mL) was added to the mixture and incubated at room temperature for 90 min. The absorbance was measured at 725 nm using a spectrophotometer (Shimadzu UV mini 1240, Duisburg, Germany). A standard curve was plotted using different concentrations of gallic acid, and the total amount of phenolics was calculated as gallic acid equivalents in µg.mL^−1^.

### 4.9. DPPH Radical Scavenging Activity

DPPH radical scavenging activity was measured according to [66] modified using Eppendorf tubes for solid fractions, microtiter plates, and a multi-plate reader. The samples were mixed in distilled water to acquire solutions with different concentrations. Afterward, 150 μL of the solution was mixed with a 150 μL 0.1 mM ethanolic solution of DPPH radical and then kept in the dark at ambient temperature for 30 min. The absorbance was read at 515 nm by an Eon™ microplate spectrophotometer (BioTek Instruments, Inc., Winooski, VT, USA). For the blank, distilled water was used instead of the sample. Control was prepared with 150 μL of sample and 150 μL of 95% ethanol. All the measurements were carried out in triplicate. A BHT solution (0.2 mg.mL^−1^) was used as a positive control. DPPH radical scavenging capacity was derived as follows:

$$\mathrm{DPPH}\;\mathrm{radical}\;\mathrm{scavenging}\;\mathrm{activity}\;(\%)\;=\;(1\;-\;\frac{\left(A_{s}-A_{c}\right)}{A_{b}})\;\times \;100$$

where *A_s_*, *A_c_*, and *A_b_* stand for absorbance of sample, control, and blank, respectively. Furthermore, sample concentrations (mg protein·mL^−1^) needed to inhibit 50% of DPPH radical activity (IC_50_ values) were determined by drawing dose-response curves.

### 4.10. Fe^2+^ Chelating Activity

Fe^2+^ chelating activity of the extracts was measured according to [67] modified using Eppendorf tubes for solid fractions, microtiter plates, and a multi-plate reader. The samples were mixed in distilled water to obtain different concentrations. Then, each extract solution (200 μL) was blended with distilled water (270 μL) plus ferrous chloride 2 mM (10 μL). The reaction was blocked after 3 min using 20 μL of ferrozine solution (5 mM). The mixture was then shaken vigorously. After 10 min at ambient temperature, the absorbance was read at 562 nm by an Eon™ microplate spectrophotometer (BioTek Instruments, Inc., Winooski, VT, USA). For the blank, distilled water was used instead of the sample. Sample control was prepared without adding ferrozine. All the measurements were carried out in triplicate. For the positive control, 0.06 mM EDTA was used. The metal chelating activity was calculated as follows:

$${\mathrm{Fe}}^{2+}\;\mathrm{chelating}\;\mathrm{activity}\;(\%)\;=\;(1\;-\;\frac{\left(A_{s}-A_{c}\right)}{A_{b}})\;\times \;100$$

where *A_s_*, *A_c_* and *A_b_* stand for absorbance of sample, control, and blank, respectively. Also, sample concentrations (mg protein·mL^−1^) needed to chelate 50% of Fe^2+^ (IC_50_ values) were determined by drawing dose-response curves.

### 4.11. Statistical Analysis

The obtained data were analyzed via analysis of variance (ANOVA), and differences between means were determined using the Tukey test. All the statistical operations were performed in OriginPro 2023 (OriginLab Co., Northampton, MA, USA). Differences were considered significant at *p* < 0.05.

## 5. Conclusions

This research demonstrated that a sequential approach, starting with aqueous extraction on *P. palmata* followed by enzymatic treatment using the resulting solid residues, effectively produces protein- and peptide-rich solid fractions with favorable antioxidant properties. Considering the low phenolic content in the solid fractions, the antioxidant properties observed may be ascribed to the proteins and peptides extracted, which contain amino acids adept at radical scavenging and metal chelation, or possibly their interactions with other macromolecules like polysaccharides. Further investigation is warranted to determine whether the highly antioxidant proteins and peptides in the solid fractions could undergo additional extraction to become soluble and potentially possess enhanced antioxidant characteristics, thereby significantly improving their practical applications. Additionally, exploring the use of solvents other than water for the initial extraction step before the enzymatic hydrolysis of the protein-rich fraction is recommended to ascertain if these solvents can more effectively expose the proteins for proteolysis. Lastly, there is considerable scope for research in testing various proteases to evaluate their effectiveness in producing peptides with antioxidant properties. Such studies would yield a more precise understanding of the extraction process for antioxidant proteins and peptides from red seaweed, affirming its viability as a sustainable source of bioactive compounds.

## Figures and Tables

**Table 1:** Amino acid (mg.g^−1^ of dry weight) contents of liquid and solid fractions after aqueous and enzymatic extraction from *P. palmata*.

	Aqueous Extraction on Seaweed						Enzymatic Extraction on Solid Fractions from Aqueous Extraction					
	Liquid Fraction (LA)			Solid Fraction (SA)			Liquid Fraction (LE)			Solid Fraction (SE)		
	pH 3	pH 6	pH 9	pH 3	pH 6	pH 9	pH 3	pH 6	pH 9	pH 3	pH 6	pH 9
Phenylalanine *	0.40 ± 0.01 ^c^	0.63 ± 0.04 ^c^	0.63 ± 0.03 ^c^	6.21 ± 0.59 ^b^	5.53 ± 0.31 ^b^	5.02 ± 0.52 ^b^	1.09 ± 0.14 ^c^	0.73 ± 0.18 ^c^	0.77 ± 0.46 ^c^	12.04 ± 1.13 ^a^	11.89 ± 1.43 ^a^	10.91 ± 0.72 ^a^
Leucine *	0.75 ± 0.04 ^c^	0.92 ± 0.11 ^c^	0.94 ± 0.09 ^c^	11.59 ± 1.30 ^b^	10.38 ± 0.50 ^b^	9.23 ± 1.01 ^b^	1.66 ± 0.23 ^c^	1.15 ± 0.32 ^c^	1.30 ± 0.52 ^c^	21.18 ± 2.28 ^a^	21.22 ± 2.58 ^a^	19.34 ± 1.62 ^a^
Isoleucine *	0.42 ± 0.03 ^c^	0.55 ± 0.06 ^c^	0.54 ± 0.06 ^c^	6.40 ± 0.69 ^b^	5.69 ± 0.37 ^b^	5.19 ± 0.62 ^b^	0.98 ± 0.16 ^c^	0.64 ± 0.18 ^c^	0.79 ± 0.34 ^c^	11.85 ± 1.14 ^a^	11.91 ± 1.68 ^a^	10.92 ± 0.92 ^a^
Methionine *	0.19 ± 0.03 ^c^	0.28 ± 0.06 ^c^	0.28 ± 0.03 ^c^	2.96 ± 0.35 ^b^	2.68 ± 0.19 ^b^	2.42 ± 0.31 ^b^	0.40 ± 0.04 ^c^	0.27 ± 0.04 ^c^	0.32 ± 0.12 ^c^	5.63 ± 0.55 ^a^	5.73 ± 0.68 ^a^	5.24 ± 0.39 ^a^
Tyrosine *	0.41 ± 0.04 ^c^	0.71 ± 0.27 ^c^	0.71 ± 0.04 ^c^	6.88 ± 0.70 ^b^	5.99 ± 0.31 ^b^	5.51 ± 0.55 ^b^	1.14 ± 0.08 ^c^	0.65 ± 0.18 ^c^	0.74 ± 0.40 ^c^	14.08 ± 0.32 ^a^	13.65 ± 0.73 ^a^	12.64 ± 0.70 ^a^
**Proline**	2.71 ± 0.05 ^c^	2.91 ± 0.29 ^c^	2.86 ± 0.10 ^c^	8.39 ± 0.85 ^b^	7.40 ± 0.29 ^b^	6.95 ± 0.77 ^b^	2.74 ± 0.11 ^c^	2.22 ± 0.28 ^c^	1.76 ± 0.55 ^c^	15.45 ± 1.48 ^a^	15.84 ± 1.66 ^a^	14.38 ± 0.86 ^a^
Valine *	1.04 ± 0.05 ^c^	1.13 ± 0.13 ^c^	1.12 ± 0.08 ^c^	12.35 ± 1.34 ^b^	10.95 ± 0.46 ^b^	10.11 ± 1.09 ^b^	2.22 ± 0.30 ^c^	1.88 ± 0.31 ^c^	1.95 ± 0.28 ^c^	24.00 ± 2.34 ^a^	23.88 ± 2.33 ^a^	21.83 ± 1.70 ^a^
**Alanine**	2.49 ± 0.15 ^c^	2.41 ± 0.32 ^c^	2.27 ± 0.28 ^c^	13.88 ± 1.28 ^b^	12.45 ± 0.67 ^b^	11.69 ± 1.30 ^b^	4.25 ± 0.62 ^c^	3.80 ± 0.29 ^c^	4.11 ± 0.24 ^c^	25.36 ± 2.32 ^a^	26.33 ± 2.94 ^a^	23.76 ± 1.53 ^a^
Threonine *	1.21 ± 0.07 ^c^	1.40 ± 0.23 ^c^	1.38 ± 0.11 ^c^	8.55 ± 1.05 ^b^	7.83 ± 0.39 ^b^	6.88 ± 1.43 ^b^	1.57 ± 0.22 ^c^	1.59 ± 0.25 ^c^	1.59 ± 0.36 ^c^	18.34 ± 1.65 ^a^	18.89 ± 1.97 ^a^	17.16 ± 1.09 ^a^
**Glycine**	2.39 ± 0.38 ^c^	3.00 ± 0.41 ^c^	2.93 ± 0.31 ^c^	13.02 ± 1.12 ^b^	11.80 ± 0.90 ^b^	10.92 ± 1.09 ^b^	3.56 ± 0.45 ^c^	2.76 ± 0.55 ^c^	2.75 ± 0.73 ^c^	22.82 ± 1.86 ^a^	23.23 ± 1.99 ^a^	21.33 ± 1.29 ^a^
**Serine**	3.12 ± 0.75 ^c^	2.62 ± 0.53 ^c^	2.59 ± 0.29 ^c^	13.20 ± 1.28 ^b^	12.32 ± 0.64 ^b^	11.18 ± 1.07 ^b^	3.11 ± 0.58 ^c^	3.35 ± 0.54 ^c^	2.91 ± 0.75 ^c^	22.58 ± 2.12 ^a^	22.74 ± 2.27 ^a^	21.32 ± 1.22 ^a^
**Arginine**	0.68 ± 0.24 ^e^	0.74 ± 0.24 ^e^	0.83 ± 0.18 ^e^	10.90 ± 0.94 ^c^	9.21 ± 0.45 ^cd^	8.24 ± 0.74 ^d^	1.03 ± 0.11 ^e^	0.99 ± 0.35 ^e^	1.06 ± 0.40 ^e^	20.39 ± 1.80 ^a^	20.37 ± 1.98 ^a^	18.10 ± 1.10 ^b^
Histidine *	ND **	ND	ND	3.31 ± 0.64 ^b^	2.76 ± 0.29 ^bc^	2.32 ± 0.30 ^c^	ND	ND	ND	5.06 ± 0.59 ^a^	5.46 ± 0.70 ^a^	4.83 ± 0.39 ^a^
**Glutamic acid**	9.52 ± 0.24 ^def^	11.80 ± 0.89 ^d^	11.03 ± 0.44 ^de^	23.09 ± 1.98 ^b^	20.24 ± 0.82 ^bc^	18.86 ± 2.15 ^c^	7.85 ± 0.41 ^ef^	6.08 ± 0.86 ^f^	6.23 ± 1.44 ^f^	35.15 ± 2.72 ^a^	35.44 ± 3.66 ^a^	32.13 ± 2.31 ^a^
Cystine *	ND	ND	ND	ND	ND	ND	ND	ND	ND	2.09 ± 0.67 ^a^	1.41 ± 0.50 ^b^	1.27 ± 0.27 ^b^
**Aspartic acid**	8.62 ± 0.67 ^d^	8.43 ± 0.62 ^d^	9.22 ± 0.43 ^d^	24.19 ± 3.10 ^b^	21.68 ± 1.30 ^bc^	20.06 ± 2.21 ^c^	5.95 ± 0.51 ^de^	4.46 ± 0.67 ^e^	5.55 ± 1.34 ^de^	36.18 ± 2.79 ^a^	37.29 ± 3.41 ^a^	34.27 ± 1.77 ^a^
TAA ***	33.95 ± 1.57 ^d^	37.51 ± 2.97 ^d^	37.34 ± 1.27 ^d^	164.92 ± 16.40 ^b^	146.90 ± 6.90 ^bc^	134.58 ± 14.70 ^c^	37.54 ± 3.57 ^d^	30.56 ± 3.30 ^d^	31.84 ± 7.19 ^d^	292.20 ± 25.73 ^a^	295.29 ± 30.84 ^a^	269.45 ± 16.81 ^a^
**EAA**	0.42 ± 0.14 ^c^	5.61 ± 0.69 ^c^	5.60 ± 0.20 ^c^	58.26 ± 6.15 ^b^	51.80 ± 2.05 ^b^	46.67 ± 5.60 ^b^	9.05 ± 1.08 ^c^	6.91 ± 1.22 ^c^	7.46 ± 2.45 ^c^	114.26 ± 10.94 ^a^	114.04 ± 13.12 ^a^	104.15 ± 7.05 ^a^
**EAA/TAA**	0.130 ± 0.002 ^d^	0.149 ± 0.009 ^d^	0.150 ± 0.005 ^d^	0.353 ± 0.004 ^b^	0.353 ± 0.003 ^b^	0.346 ± 0.004 ^b^	0.241 ± 0.006 ^c^	0.224 ± 0.016 ^c^	0.230 ± 0.029 ^c^	0.391 ± 0.005 ^a^	0.386 ± 0.005 ^a^	0.386 ± 0.003 ^a^

Data were expressed as mean ± standard deviation. Superscripts denote significant differences among the treatments (*p* < 0.05). LA and SA denote liquid and solid fractions after aqueous extraction, respectively; LE and SE stand for liquid and solid fractions after enzymatic extraction (using the solid fraction from aqueous stage as substrate), respectively. * Essential amino acids (EAA) in human nutrition [19]. ** Not Detected. *** Total amino acids.

**Table 2:** In vitro antioxidant properties of liquid and solid fractions after aqueous and enzymatic extraction from *P. palmata*.

Extraction Method	pH Value	IC_50_ (mg.mL^−1^) for DPPH Radical Scavenging Activity		IC_50_ (mg.mL^−1^) for Fe^2+^ Chelating Activity	
		Liquid Fraction	Solid Fraction	Liquid Fraction	Solid Fraction
**Aqueous extraction on seaweed (LA and SA)**	3	9.31 ± 0.19 ^c^	3.97 ± 0.07 ^a^	NR *	4.81 ± 0.05 ^bc^
	6	9.03 ± 2.72 ^c^	2.85 ± 0.08 ^a^	5.06 ± 0.55 ^c^	11.84 ± 2.50 ^ef^
	9	NR	8.15 ± 0.02 ^bc^	1.26 ± 0.07 ^a^	8.97 ± 0.36 ^de^
**Enzymatic extraction on solid fraction from aqueous extraction (LE and SE)**	3	10.41 ± 0.51 ^c^	2.29 ± 1.00 ^a^	5.52 ± 0.60 ^cd^	0.63 ± 0.04 ^a^
	6	10.09 ± 0.57 ^c^	2.92 ± 0.02 ^a^	14.60 ± 0.15 ^f^	0.89 ± 0.07 ^a^
	9	NR	4.38 ± 0.17 ^ab^	8.92 ± 1.26 ^de^	1.35 ± 0.11 ^ab^

Data were expressed as mean ± standard deviation. Superscripts denote significant differences in each column (*p* < 0.05). LA and SA denote liquid and solid fractions after aqueous extraction, respectively; LE and SE stand for liquid and solid fractions after enzymatic extraction (using the solid fraction from aqueous stage as substrate), respectively. * Not Reached (The sample could not scavenge at least 50% of DPPH radical or could not chelate at least 50% of Fe^2+^ at the maximum concentration tested, i.e., 16 mg.mL^−1^).

## Data Availability

The data acquired in this study can be obtained on request.

## List of Abbreviations

ANOVA	analysis of variance
BHT	butylated hydroxytoluene
DH	degree of hydrolysis
DPPH	1,1-diphenyl-2-picrylhydrazyl
DTT	dithiothreitol
EAA	essential amino acids
EDTA	ethylenediaminetetraacetic acid
HCl	hydrochloric acid
HPLC	high-performance liquid chromatography
IC_50_	the concentration needed to inhibit free radicals or chelate metal ions by 50%
LA3	liquid fraction obtained after aqueous extraction at pH 3
LA6	liquid fraction obtained after aqueous extraction at pH 6
LA9	liquid fraction obtained after aqueous extraction at pH 9
LAPU	leucine aminopeptidase units
LE3	liquid fraction obtained after enzymatic extraction at pH 3
LE6	liquid fraction obtained after enzymatic extraction at pH 6
LE9	liquid fraction obtained after enzymatic extraction at pH 9
LFPC	liquid fraction’s protein content
LFPR	liquid fraction’s protein recovery
OPA	o-phthaldialdehyde
Na_2_CO_3_	sodium carbonate
NaHCO₃	sodium bicarbonate
NaOH	sodium hydroxide
SA3	solid fraction obtained after aqueous extraction at pH 3
SA6	solid fraction obtained after aqueous extraction at pH 6
SA9	solid fraction obtained after aqueous extraction at pH 9
SE3	solid fraction obtained after enzymatic extraction at pH 3
SE6	solid fraction obtained after enzymatic extraction at pH 6
SE9	solid fraction obtained after enzymatic extraction at pH 9
SFPC	solid fraction’s protein content
SFPR	solid fraction’s protein recovery
TAA	total amino acids
TPC	total phenolic content
